# An approach to predict the risk of glaucoma development by integrating different attribute data

**DOI:** 10.1186/2193-1801-1-41

**Published:** 2012-10-24

**Authors:** Yuichi Tokuda, Tomohito Yagi, Kengo Yoshii, Yoko Ikeda, Masahiro Fuwa, Morio Ueno, Masakazu Nakano, Natsue Omi, Masami Tanaka, Kazuhiko Mori, Masaaki Kageyama, Ikumitsu Nagasaki, Katsumi Yagi, Shigeru Kinoshita, Kei Tashiro

**Affiliations:** 1 Department of Genomic Medical Sciences, Kyoto Prefectural University of Medicine, Kajiicho 465, Kawaramachi-Hirokoji, Kamigyo-ku, Kyoto, 602-8566 Japan; 2 Department of Ophthalmology, Kyoto Prefectural University of Medicine, Kajiicho 465, Kawaramachi-Hirokoji, Kamigyo-ku, Kyoto, 602-8566 Japan; 3 Research and Development Center, Santen Pharmaceutical Co. Ltd, 8916-16 Takayama-cho, Ikoma, Nara, 630-0101 Japan; 4 Department of Mathematics, Kyoto Prefectural University of Medicine, Kajiicho 465, Kawaramachi-Hirokoji, Kamigyo-ku, Kyoto, 602-8566 Japan; 5 Louis Pasteur Center for Medical Research, 103-5, Tanakamonzen-cho, Sakyo-ku, Kyoto City, Kyoto, 606-8225 Japan

**Keywords:** *Glaucoma*, GWAS, Machine learning, Integration approach

## Abstract

Primary open-angle glaucoma (POAG) is one of the major causes of blindness worldwide and considered to be influenced by inherited and environmental factors. Recently, we demonstrated a genome-wide association study for the susceptibility to POAG by comparing patients and controls. In addition, the serum cytokine levels, which are affected by environmental and postnatal factors, could be also obtained in patients as well as in controls, simultaneously. Here, in order to predict the effective diagnosis of POAG, we developed an “integration approach” using different attribute data which were integrated simply with several machine learning methods and random sampling. Two data sets were prepared for this study. The one is the “training data set”, which consisted of 42 POAG and 42 controls. The other is the “test data set” consisted of 73 POAG and 52 controls. We first examined for genotype and cytokine data using the training data set with general machine learning methods. After the integration approach was applied, we obtained the stable accuracy, using the support vector machine method with the radial basis function. Although our approach was based on well-known machine learning methods and a simple process, we demonstrated that the integration with two kinds of attributes, genotype and cytokines, was effective and helpful in diagnostic prediction of POAG.

## Introduction

Glaucoma is a progressive eye disease that shows characteristic degeneration of the optic nerve and visual field defects (Kwon et al. 2009). Among the subtypes of glaucoma, primary open-angle glaucoma (POAG) is a major cause of blindness worldwide. The results of many studies have suggested that a genetic contribution is one of the risk factors for the development of glaucoma (Ray & Mookherjee 2009). However, it is still unclear if the genetic risk factors contribute to all of the pathogenesis of glaucoma. To investigate the mechanism(s) of common diseases such as glaucoma, genome-wide association studies (GWAS) have been widely performed (Consortium TWTCC 2007; Balding 2006). GWAS is one of the powerful tools to identify genetic association to common diseases with genotype data for single nucleotide polymorphisms (SNPs). Previously, we performed a GWAS to identify the common POAG-associated genetic factors (Nakano et al. 2009) and found a number of SNPs significantly associated with POAG. GWAS for POAG has also been performed by several other research groups (Meguro et al. 2010; Thorleifsson et al. 2010; Burdon et al. 2011), and we also recently published additional GWAS research results on POAG (Nakano et al. 2012). However, compared with the genetic risk for another type of glaucoma, Exfoliation Glaucoma (EG), which was carried out by deCODE using only two SNPs (http://www.decodehealth.com/glaucoma), genetic contribution for POAG seems to be a complex. In EG, SNPs were highly significant on a single gene, LOXL1, by GWAS (Thorleifsson et al. 2007; Williams et al. 2010; Mabuchi et al. 2008; Fan et al. 2008), while in POAG, several genes are involved as genetic risk factors. In addition, besides the genetic factor, POAG is considered to have other risk factors (Kwon et al. 2009) as well. Thus, precise disease mechanism(s) of POAG remains elusive.

For the purposes of diagnostic prediction or finding out the pathogenesis of diseases, genotype data have been applied in several machine-learning algorithms (Relton et al. 2004; Listgarten et al. 2004; Ritchie et al. 2001; Nelson et al. 2001; Hoh et al. 2001; Wang et al. 2012). Genetic data and the other risk factors (e.g., smoking, body mass index) were combined for these prediction models (Seddon et al. 2009). In such studies, careful extraction of attributes for prediction from large volumes of data and appropriate data selection from several attributes are essential. As the development of common diseases like POAG is influenced by many factors, the contribution of each attribute weighs variously among the patients. Thus, for the diagnostic prediction of POAG, clarification of each attribute obtained for analysis needs to be carefully assessed. In this regard, it is important to develop a new strategy of integrating the data with various attributes for establishing useful diagnostic prediction.

In order to evaluate the risk factor of POAG, we integrated cytokine data together with genetic data as a new strategy. We focused on the serum cytokines because the relation between glaucomatous neurodegeneration and immune response was previously suggested (Tezel 2011), and several cytokines were reported to be linked with glaucoma (Huang et al. 2010; Yang et al. 2001). Cytokines, which include both chemokines and lymphokines, are small soluble proteins that play a pivotal role in immune system. The concentration of serum cytokines may reflect the physiological condition of the hosts affected by environmental and postnatal factors as one of the important indices useful for the diagnostic prediction of certain diseases. Obviously, cytokine data as an attribute weigh differently from those of the genotype data. In addition, the equipments that many cytokines can measure simultaneously under the same condition could have been developed and applied to diagnostic analysis (Ray et al. 2007; Lambeck et al. 2007). Therefore, we especially tried to measure and handle many cytokines simultaneously.

Here, for predicting the risk of POAG development, we attempted to establish a new integration approach with a good potential as a useful and simple tool. This procedure performs the integration of data with various kinds of attributes by using several machine learning methods with random sampling. In particular, because both genotyping and cytokines attributes were obtained from blood sample, our approach is considered to be useful for assessment of the risk of POAG and predicting the onset possibility before consulting ophthalmologists. This strategy may give us with new prototype for a clinical approach in understanding the underlying mechanism(s) of various diseases, not limited to POAG.

## Methods

### Sample Information

To obtain the peripheral blood samples, 115 POAG patients and 94 healthy control volunteers were recruited at the University Hospital of Kyoto Prefectural University of Medicine (Kyoto, Japan). This study was approved by the institutional review board of Kyoto Prefectural University of Medicine and conducted in accordance with the principles set forth in the Helsinki Declaration. All participants were interviewed about their familial history of glaucoma and other diseases and diagnosed either POAG or control by three ophthalmologists (YI, MU, and KM). The 115 POAG patients had peak intraocular pressure ≥ 22 mmHg without treatment. Peripheral blood samples were collected simultaneously from each participant for obtaining genomic DNA for genotyping and serum for cytokine measurement. DNA and sera were stored at −80°C until examined.

These samples were divided into two groups, since the cytokine data was obtained with two conditions. The first was defined as the “training data set” and the other as the “test data set” (Table 1). The former consisted of 42 POAG and 42 healthy control samples and was utilized in the training process of the machine learning. The latter consisted of 73 POAG and 52 healthy control samples, which were applied for the diagnostic prediction of POAG.

**Table 1:** **Clinical characteristic of samples**

	Training data set		Test data set	
	POAG	Control	POAG	Control
Number of sample	42	42	73	52
Famale / male ratio	1.00	0.83	0.62	1.74
Age at blood sampling	56.4±5.5	55.3±3.4	70.9±10.7	61.8 ± 11.3
Storage period of blood (days)	880.1±112.0	865.7±106.0	1044.0±114.4	892.2 ± 129.9

### Genotype data

All genotype data were obtained by GeneChip® Human Mapping 500K Array platform (Affymetrix) according to the manufacturer’s instructions. Although this array system carries the probes for more than five hundred thousand SNPs, we needed a number of SNPs significantly associated with POAG for the tests. Our previous study (Nakano et al. 2009) suggested that 40 SNPs were significantly POAG-associated which had both Mantel-Haenszel p-value of less than 0.01 and a p-value of Cochran’s Q test (Ioannidis et al. 2007) equal to or more than 0.05 in the two stage GWAS. Because the pairs of SNPs showing high linkage disequilibrium (LD) could cause a multicollinearity problem, the Haploview program (Barrett et al. 2005) was applied to calculate LD. As a result, 11 of the 40 SNPs were excluded because of their high LD and remaining 29 SNPs were employed in this study (Table 2). All of the genotype data except for the missing by genotyping failure, which were represented by a pair of letters (e.g., AA, AT and TT), were converted into discrete numerical values according to the number of allele with higher frequency in the POAG (i.e., risk allele) as followed: risk allele homozygote, 2; risk allele heterozygote, 1; and other allele homozygote, 0. Then, all the genotype data were normalized using the equations in EIGENSTRAT (Price et al. 2006), so that the missing data were set to 0.0. According to the allele frequency and the average of numeric genotypes calculated from the training data set, this normalization was carried out and the normalized data represented discrete values.

**Table 2:** **Summary of 29 SNPs used in this study**

dbSNP ID	Chr.	SNP type	Nearest gene	Genotype frequency
rs547984	1	intergenic	ZP4	AA(0.263) AC(0.488) CC(0.249)
rs1892116	1	intronic	AHCTF1	AA(0.507) AG(0.445) GG(0.048)
rs4666488	2	intergenic	OSR1	AA(0.100) AG(0.397) GG(0.503)
rs2268794	2	intronic	SRD5A2	AA(0.005) AT(0.319) TT(0.676)
rs7574012	2	intergenic	QPCT	AA(0.373) AG(0.459) GG(0.168)
rs1990702	2	intergenic	LRP2	GG(0.120) GA(0.433) AA(0.447)
rs10930437	2	intergenic	SP5	AA(0.429) AG(0.454) GG(0.117)
rs779701	3	intronic	GRM7	AA(0.490) AG(0.413) GG(0.097)
rs6550783	3	intergenic	UBE2E1	AA(0.412) AG(0.442) GG(0.146)
rs6550308	3	intergenic	ARPP21	GG(0.215) GA(0.488) AA(0.297)
rs3922704	3	intronic	PLCXD2	CC(0.034) CG(0.254) GG(0.712)
rs17279573	4	intergenic	KIAA0922	GG(0.120) GA(0.483) AA(0.397)
rs818725	5	intronic	ADAMTS12	CC(0.019) CG(0.226) GG(0.755)
rs11750584	5	intergenic	HEATR7B2	CC(0.029) CG(0.292) GG(0.679)
rs9640055	7	intronic	GLCCI1	GG(0.038) GA(0.344) AA(0.618)
rs2966712	7	intergenic	LOC285965	AA(0.005) AG(0.211) GG(0.784)
rs411102	9	intergenic	KRT8P11	GG(0.749) GA(0.242) AA(0.009)
rs7850541	9	intergenic	GBGT1	GG(0.514) GA(0.361) AA(0.125)
rs7081455	10	intergenic	PLXDC2	AA(0.644) AC(0.293) CC(0.063)
rs493622	11	intergenic	CHORDC1	AA(0.565) AC(0.383) CC(0.052)
rs610160	11	intronic	GRIA4	AA(0.693) AG(0.262) GG(0.045)
rs7961953	12	intronic	TMTC2	GG(0.522) GA(0.397) AA(0.081)
rs10492680	13	intergenic	FLJ42392	GG(0.005) GA(0.187) AA(0.808)
rs1571379	14	intergenic	SEL1L	AA(0.440) AG(0.454) GG(0.106)
rs9788983	17	intronic	RPH3AL	AA(0.770) AG(0.215) GG(0.015)
rs16940484	18	intronic	TTC39C	GG(0.469) GA(0.450) AA(0.081)
rs2864107	19	intergenic	ZNF175	GG(0.684) GA(0.301) AA(0.015)
rs6115865	20	intergenic	C20orf194	AA(0.125) AG(0.428) GG(0.447)
rs5765558	22	intergenic	ATXN10	AA(0.287) AG(0.478) GG(0.235)

The dbSNP ID represents with build 130. Chr. denotes the number of chromosome. The Nearest genes are positioned nearest by each SNP and referred to NCBI Build 36. Genotype frequencies are calculated by total samples used in this study, which are 115 POAG patients and 94 healthy control volunteers.

### Cytokine data

Serum cytokines were measured by the bead flow-cytometry analysis by the Becton Dickinson (BD, San Diego, CA) Cytometric Bead Array (CBA™) Flex Set System according to the manufacturer’s protocol. The data was examined by a BD FACSArray™ (BD) flow cytometer with FCAP Array™ software and the BD FACSArray™ Bioanalyzer (BD).

In this study, we first assayed 29 cytokines in the sera from “the training data set”, and each cytokine concentration was calculated from each raw data by the Four Parameter Logistic Model (FPLM), which was recommended by the manufacturer (http://www.bdbiosciences.com/documents/Analysis_of_data_from_CBA_using_FCAPArray.pdf). Before we performed the statistical analysis, the quality of the cytokine data was evaluated. Of 29 cytokines, 21 cytokines were excluded; 7 were for measurement failures (over 5% of the 84 samples) and 14 for concentration of zero (over 5% of the 84 samples). The remaining 8 cytokines were tested by the Student’s *t*-test between the POAG and control samples, of which 5 cytokines were excluded with a p-value over 5%. Eventually, only 3 cytokines, i.e., Fas Ligand, Eotaxin, and MIG, were picked up to be significantly associated with POAG from the training data set samples (Table 3).

**Table 3:** **Summary of the three cytokines used in the integration approach**

Cytokine			Training data set			Test data set	
			Concentration	P-value^*^		Concentration	P-value^*^
Fas Ligand		POAG	63.5 (52.2-87.3)	0.002		37.5 (31.8-46.6)	0.877
		Control	53.3 (34.9-63.4)			36.2 (28.0-45.4)	
Eotaxin		POAG	309.1 (273.6-342.9)	0.038		70.6 (54.9-90.8)	0.013
		Control	268.5 (236.7-311.6)			63.5 (54.4-73.9)	
MIG		POAG	410.9 (306.8-524.9)	0.021		318.1 (182.9-511.7)	0.109
		Control	340.4 (198.9-470.1)			148.4 (117.7-241.9)	

“Concentration” represents the median concentration and interquartile range. ^*^ P-value of the comparison between POAG and control calculated by Student’s *t*-test.

Subsequently, these 3 cytokines were determined with the same assay procedure on 126 samples (73 POAG and 53 controls) from the “test data set” samples. Data were obtained from 125 samples, excluding one control sample of failed assay (Table 3). For statistical analysis, the cytokine concentration data were standardized in order to minimize the biases among the assay conditions as followed. Let *c*_*ij*_ be the cytokine concentration measured for cytokine *i* and sample *j*, where *i* = 1 to 3 and *j* = 1 to *M* (*M* is 84 in the training data set; 125 in the test data set). Let *m*_*i*_ and *s*_*i*_ be the mean and standard deviation of cytokine *i*, respectively. At each data set, *m*_*i*_ and *s*_*i*_ were calculated only for the control samples because it was considered that the cytokine concentration of healthy control samples might act fairly consistently under each experimental condition. The standardized value *n*_*ij*_ was calculated using the following equation: *n*_*ij*_ = (*c*_*ij*_ - *m*_*i*_)/*s*_*i*_. Notably, the cytokine concentration data was obtained as continuous values when they were calculated by FPLM.

Finally, results of a total of 32 attributes, which consisted of 29 SNPs (Table 2) and 3 cytokines (Table 3), were applied for “integration approach” in this study.

### Base classifiers

In this study, well-known machine learning methods, i.e., Linear Discriminant Analysis (LDA), Support Vector Machine (SVM), Naive Bayes Classifier (NBC), and Decision Tree (DT) were applied. We defined these methods as “base classifiers”.

LDA is a method used in statistics and machine learning to find a discriminant function by which two or more groups can be separated. LDA seeks a linear function of the variables (e.g., genotype and cytokine) in the training data set that maximizes the distance among means in each group as it minimizes the within-group variance. Hence, a discriminant function can be computed explicitly and used as a linear classifier.

SVM is a supervised machine learning method based on the idea of classifying two groups by a hyperplane with a large margin. SVM maps the data in the training data set into a possibly higher dimension of space by using a kernel function. In the space, SVM learns the classifier by seeking a hyperplane that may separate the two groups by a certain distance. If the training data set is not separated linearly, SVM optimizes the separation between the two groups. The kernel function in SVM is decided according to the attribute of the data. In this study, we used SVM for learning with three kernel functions: linear, polynomial, and radial basis function (RBF).

NBC is a simple and efficient probabilistic classifier based on Bayes’ theorem. Assuming there is independence between each set of attribute data (e.g., genotype or cytokine); NBC calculates the probabilities used for the prediction from the training data set. As each sample in the test data set is given to the NBC, it predicts to which group (e.g., POAG or control) the sample belongs by the highest conditional probability.

DT is a tree-like data structure used for learning a method to classify data hierarchically by sequential decision process. Basically, DT is a binary tree and each node splits the data by each feature (i.e., large/small, male/female). In this study, DT was performed by CART (Classification and Regression Trees), and used to classify SNPs (each data consisted four discrete; three genotypes and missing data) and cytokines (each data was continuous).

All the data analysis and drawing figures were performed with R software (version 2.14.0) (R Development Core Team 2011); the LDA was implemented by the MASS (version 7.3-16) R package; the SVM and NBC functions were implemented by the e1071 (version 1.6) R package (Dimitriadou et al. 2011); and the DT functions were implemented by the mvpart (version 1.4-0) R package. In addition, each classifier was performed with default parameter settings.

Accuracy, sensitivity and specificity of the data (genotype and cytokine) for the POAG prediction were calculated by these analytical procedures.

### Integration approach

In this study, the data consists of two kinds of attributes in that the genotype data are discrete and the cytokine data are continuous. In most cases, it is easy and no problem to apply these data for each method simply and simultaneously. However, one must be careful to integrate them while considering each attribute, especially to note how each attribute contributes. The prediction may be made possible from analytical results for each type of attribute data instead of applying the data directly, because of the difference in the attributes. In addition, if the analytical results show differences between each attribute, the prediction for each sample has interesting information how each attribute contributes. For these reasons, we performed the integration approach so that after the genotype and cytokine data are separately applied in the processes, their results are integrated after the last process. To enable an effective analysis by integrating these two kinds of data, this approach is based on the idea of ensemble learning (e.g., Bootstrap aggregating (Bagging) (Breiman 1996)). Bagging is one of the powerful prediction tools for improving other basic classifier. For example, bagging is used for the purpose of improving the diagnosis of Valvular Heart Disease by SVM (Sengur 2012), or assessing the interactions of SNPs (Schwender et al. 2011).

For the training data set *L* consisted of cases (*l*^*P*^_1_,…,*l*^*P*^_*p*_) and controls (*l*^*c*^_1_,…,*l*^*c*^_*q*_) and the test data set *T* = {*t*_1_,…,*t*_*r*_}, the integration approach consists of the following steps:

1. Obtain *Sg*, which is the subset of the training data set, by random sampling without replacement from *L* so that the same number of samplings is taken from the cases as from the controls.
2. Apply the base classifiers to the genotype data of *Sg* to obtain a predictor *Pg* as a training result.
3. Repeat above steps (1) and (2) *K* times; this process produces genotype data predictors {*Pg*_1_,…,*Pg*_*K*_} from {*Sg*_l_,…,*Sg*_*K*_}.
4. In addition, repeat the same process as in (1) and (2) above *N* times for cytokine data; cytokine data predictors {*Pc*_1_,…,*Pc*_*N*_} are produced from the subset of the training data set {*Sc*_l_,…,*Sc*_*N*_}.
5. For each *t*_*j*_ in the test data *T*, the predictor gives a result which predicts whether *t*_*j*_ belongs to the cases (positive) or the controls (negative). Thus for each *t*_*j*_ in the test data *T*, the genotype data predictors {*Pg*_1_,…,*Pg*_*K*_} produce *K* prediction results {*Rg*_1_,…,*Rg*_*K*_} and the cytokine data predictors {*Pc*_1_,…,*Pc*_*N*_} produce *N* prediction results {*Rc*_1_,…,*Rc*_*N*_}.
6. For each *t*_*j*_ in the test data *T*, the majority vote of the *N* + *K* prediction results is the final prediction for *t*_*j*_.

This procedure adopted the same number of samplings, for example, 20 POAG and 20 healthy controls were sampled from 42 POAG and 42 healthy controls in the training data set, respectively. This reason is that the contribution of the characteristics of POAG and control should be as close to equal possible. Besides, it is preferable for the genotype and cytokine data to be evaluated as equally as possible (e.g., *K* = *N*.) However, it may be impossible to predict one group by dividing it in half if the total number of sampling repeats is an even number. In this study, since the size of the genotype data set was greater than that of the cytokines, *K* is taken as *N* + 1 to avoid the situation of a tie vote. In addition, note that use of the base classifier should be limited to one kind of classifier from the beginning of this procedure to the end.

## Results

### Single classifier analysis

Single classifier analysis was performed for each base classifier on 29 SNPs and 3 cytokines each and both integrated (Table 4). All of these tests were first done by the training data set and evaluated to predict the test data set. Except for DT, the accuracy of genotype data prediction was higher than that of cytokines for each base classifier. The integrated accuracy was better than each base classifier, when tested with use of the polynomial SVM, RBF SVM, and NBC. However, the integrated sensitivity (0.521) was lower than the genotype (0.589) or cytokine (0.658) prediction alone, when tested by polynomial SVM, in spite of increasing the integrated specificity (0.846) from the genotype (0.731) or cytokine (0.308) prediction alone. By contrast, RBF SVM test increased all of the accuracy (0.744), sensitivity (0.767) and specificity (0.712) on the integrated data from either genotype or cytokine prediction. These results suggested that both genotype and cytokine attributes contributed, especially when integrated, to improve the diagnostic prediction based on the base classifier.

**Table 4:** **Summary of the three cytokines used in the integration approach**

Base classifier			Single analysis			Analysis with sampling^*^		
			Accuracy	Sensitivity	Specificity	Accuracy	Sensitivity	Specificity
LDA		Genotype	0.688	0.712	0.654	0.671 ± 0.011	0.693 ± 0.015	0.639 ± 0.014
		Cytokine	0.592	0.466	0.769	0.584 ± 0.010	0.457 ± 0.012	0.763 ± 0.010
		Integrated	0.632	0.616	0.654	0.655 ± 0.022	0.611 ± 0.034	0.717 ± 0.015
SVM	linear	Genotype	0.664	0.699	0.615	0.683 ± 0.013	0.754 ± 0.023	0.584 ± 0.016
		Cytokine	0.568	0.452	0.731	0.577 ± 0.008	0.458 ± 0.012	0.745 ± 0.013
		Integrated	0.659	0.648	0.673	0.668 ± 0.014	0.640 ± 0.024	0.706 ± 0.012
	polynomial	Genotype	0.648	0.589	0.731	0.633 ± 0.010	0.539 ± 0.026	0.764 ± 0.018
		Cytokine	0.512	0.658	0.308	0.457 ± 0.012	0.275 ± 0.077	0.713 ± 0.086
		Integrated	0.656	0.521	0.846	0.624 ± 0.010	0.480 ± 0.065	0.827 ± 0.078
	RBF	Genotype	0.688	0.712	0.654	0.676 ± 0.010	0.685 ± 0.016	0.664 ± 0.013
		Cytokine	0.648	0.712	0.558	0.662 ± 0.006	0.701 ± 0.011	0.607 ± 0.020
		Integrated	0.744	0.767	0.712	0.740 ± 0.013	0.805 ± 0.020	0.650 ± 0.014
NBC		Genotype	0.640	0.671	0.596	0.630 ± 0.006	0.651 ± 0.013	0.601 ± 0.014
		Cytokine	0.624	0.479	0.827	0.621 ± 0.006	0.489 ± 0.013	0.807 ± 0.019
		Integrated	0.744	0.767	0.712	0.698 ± 0.013	0.644 ± 0.027	0.775 ± 0.051
DT		Genotype	0.536	0.342	0.808	0.562 ± 0.025	0.411 ± 0.070	0.774 ± 0.043
		Cytokine	0.624	0.904	0.231	0.605 ± 0.018	0.874 ± 0.099	0.226 ± 0.126
		Integrated	0.600	0.959	0.096	0.617 ± 0.013	0.668 ± 0.032	0.545 ± 0.040

^*^These values are represented as the mean and SD of each statistics. The mean of each statistics included extremely good or bad result, especially small sampling size and few sampling repeat time.

### Integration approach analysis

The results of single use with base classifier demonstrated fluctuations on each or both applying attribute (Table 4; Single analysis). Therefore, the further integrated approach was performed using each base classifier by changing the size and time of parameters (Table 4; Analysis with sampling). One of the changed parameters was the size of the subset sampling from the training data set (defined as “sampling size”), and the other was the sampling repeat times (defined as “sampling time”). The sampling size was increased from 40 (consisted of 20 POAG and 20 healthy controls) to 80 (consisted of 40 POAG and 40 healthy controls) with an equal number of samples from POAG and controls. (i.e., 21 steps were tested) On the other hand, the sampling time for each genotype and cytokine was also increased from 25 to 1,500 by 60 steps. (i.e., 25, 50, 75, ···, 1,450, 1,475 and 1,500 repeat times were tested) Moreover, because the sampling time for the genotype data was increased by one, the total sampling repeat times increased from 51 to 3,001. As a result, the integration approach was performed on 1,260 tests (21 steps of sampling sizes × 60 steps of sampling times) per each base classifier.

These results are summarized in “Analysis with sampling” in Table 4. The LDA, Linear SVM, and DT methods improved the mean of integrated accuracy from single analysis (from 0.632 to 0.655, from 0.659 to 0.668, and from 0.600 to 0.617, respectively), although those values included fluctuations due to parameter settings. The mean of the integrated accuracy (0.740 ± 0.013; mean ± SD) assessed by the RBF SVM method was the best results in analysis with sampling, however, it was slightly lower than that in single analysis in association with the higher integrated sensitivity (0.805 ± 0.020) than that in single analysis (0.767). Moreover, the specificities of genotype (0.664 ± 0.013) and cytokine (0.607 ± 0.020) by SVM RBF method in analysis with sampling were better than those in single analysis (0.654 and 0.558, respectively). In addition, some accuracy in the 1,260 tests was achieved over the single analysis.

**Figure 1:**
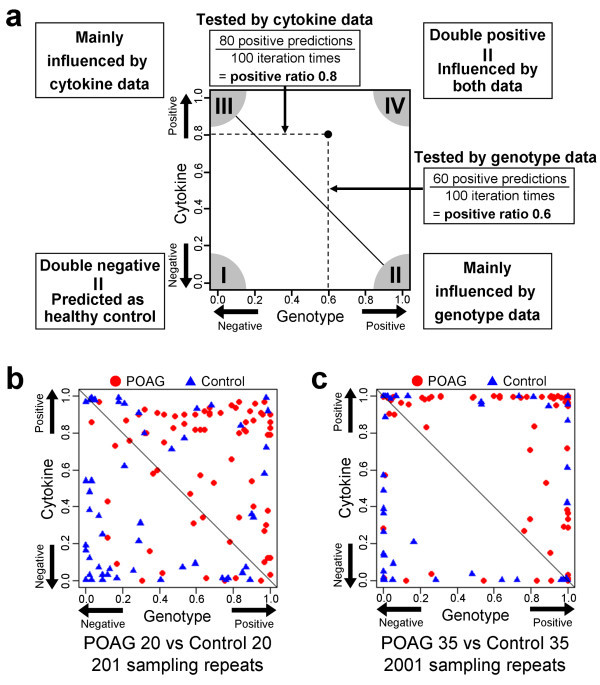
**Scatter plot showing the ratio of POAG prediction for each sample.** Figure 1 (**a**) The example figure for the scatter plot. The horizontal axis represents the ratio of positive prediction using genotype data. The positive prediction indicated the sample with POAG feature, and the negative prediction indicated the sample with control feature. The ratio was obtained by dividing the number of positive predictions by the total test number. Thus, “1” and “0” indicate 100% prediction as positive and negative, respectively. The vertical axis similarly represents the ratio using the cytokine data. Dots and triangles represent POAG and control samples, respectively. The figure can be read as, if one POAG sample was predicted as positive 60 times using the genotype data and 80 times using the cytokine data each with 100 sampling repeat times, the sample is plotted at (0.6, 0.8) by dot. If the approach has a good performance (means; highly negative or positive prediction) for samples with interaction between those two attributes, more samples will be plotted in the corner I or corner IV. If either the genotype or cytokine data is at risk for POAG, such samples will be plotted in the corner II or corner III, respectively. The diagonal line shows the threshold of the prediction by the integration approach. If a sample is plotted above or below the threshold, the final prediction result is positive or negative, respectively. Figure 1 (**b**) shows one of the examples as the comparatively smaller and unstable, which is the result with 40 sampling size and 201sampling times by RBF SVM method. Figure 1 (**c**), one of the examples as the best stable result, which is the result with 70 sampling size and 2,001sampling times by RBF SVM method.

In order to understand how the test results improved by changing the sampling size and time of parameters and each attribute contributed to the prediction, the integration results were demonstrated graphically (Figure 1). The schematic presentations of the genotype and cytokine data were plotted on horizontal and vertical axes, respectively, as shown in Figure 1a. One example of the unstable results was shown in Figure 1b. Because those parameters were comparatively smaller, the positive ratios of each attribute were generally unsatisfactory with several samples being plotted in the vicinity of the diagonal threshold. By contrast, when the sampling size was 70 (consisted of 35 POAG and 35 healthy controls) and sampling times was 2,001 (1,001 times at genotype data and 1,000 times at cytokine data), most of the samples were plotted in the vicinity of the axes (Figure 1c). Using these parameters, the accuracy was improved for 0.768. This result was also obtained by many other conditions when the sampling size and time were comparatively larger; therefore it was considered as the best stable results of the integration approach. Thus, the predictions were improved by changing the size and time of parameters in either the genotype or cytokine test.

In these test plot presentations, we focused on the contribution of the genotype and cytokine data to the stable results among the POAG samples, 23 (31.5%) showed more than 90% accuracy for both positive ratios (i.e., plotted in the corner IV in Figure 1c). On the other hand, 14 (26.9%) of the control samples showed more than 90% accuracy (i.e., plotted in the corner I in Figure 1c).

## Discussion

Bootstrap methods, such as Bagging (Breiman 1996), are generally applied in approaches using random sampling techniques. In a typical procedure, bootstrap can provide us with an estimated distribution for statistical analysis by random sampling with replacement from all samples in the data set. In this study, the method of random sampling was independent for each group, and an equal number of samples were adopted in order to avoid bias by the difference in sample numbers among each group. Additionally, our approach adopted random sampling without replacement due to the potential for multicollinearity. Because genotype data show discrete values consisted of three genotypes and one missing data, the combinations of values were easy to be limited as much as causing multicollinearity. Especially, this phenomenon was apparent when LDA method was applied with the small sampling size. For this reason, the changing parameters of the sampling size were started with 40 samples by random sampling without replacement. Besides, the accuracy did not improve without any relation to the iteration times even when the sampling size was increased enough as showed in Figure 1c. This tendency was considered to be caused by highly correlated samples. To solve this problem, it might be better to adopt the data for random sampling with replacement than without replacement according to the size of the training data set.

Using genotype data, the diagnostic prediction of POAG by RBF SVM method generally performed well also in our study (Ban et al. 2010; Rojas et al. 2009). The applied 29 SNPs were selected by the statistical result of GWAS from enormous genotype data. Employment of the SNPs selected by some large size of population was useful for this type of diagnostic prediction study without complex procedures. Thus, simple strategy might be suitable for the post GWAS analysis. The bagging is generally considered to reduce variance of classifier such as DT method; therefore, the classifier with less variant such as SVM method was considered to be improved a little by bagging. However the result of our study was effective even when SVM, DT methods with bagging was not improved.

Using cytokine data, the diagnostic prediction of POAG by RBF SVM method also performed well, regardless of some fluctuation between two data sets. Thus, RBF SVM method was thought to be successfully suitable for each attribute data, genotype as well as cytokine, in our study. In other words, the base classifier is necessary to select suitably according to each attribute. However, the effectiveness of cytokine data analysis using SVM has been reported for selecting the significant cytokines to elucidate the pathway of inflammatory response (McKinney et al. 2006).

In this study, we found 3 cytokines that are associated with POAG in 29 cytokines. In our approach, some samples was certainly predicted by only cytokine attributes as shown in Figure 1b or c. These results demonstrated that POAG patients with low genetic risk were predicted by cytokine attributes effectively.

In terms of the integration approach, one of our goals is to predict the diagnosis and/or prognosis by the patterning of different types of experimental data. In the process, an interaction between genotype and cytokine might indicate a risk of disease development, because approximately 30% of the samples in the test data set were performed with a high prediction from both types of data. Our approach also elicited a good classification of same sample when one of the two data sets was used individually before integrating them. The classification was made successful by using one data set because either genotype or cytokine behaved as a risk of disease development in these samples. For such reasons, our approach is considered to be one of the good tools to analyze the mixed data, irrespective of their interaction.

In conclusion, we demonstrated that our integration approach improved the diagnostic prediction of POAG with use of two attributes, SNPs as genotype and serum cytokines. Although two attribute data are applied independently, this approach is not affected by the differences of attribute, because the base classifier was first set according to each type of attribute data. It was confirmed that when the setting of the base classifier for one data set is successfully optimized, the integration approach might be applied using additional data with other attributes. In view of the versatility and simplicity, our approach was thought to be effective and useful for various clinical applications in future.
